# SERMs Promote Anti-Inflammatory Signaling and Phenotype of CD14+ Cells

**DOI:** 10.1007/s10753-018-0763-1

**Published:** 2018-03-24

**Authors:** Lauri Polari, Anu Wiklund, Sofia Sousa, Lauri Kangas, Tero Linnanen, Pirkko Härkönen, Jorma Määttä

**Affiliations:** 1 Institute of Biomedicine, University of Turku, Turku, Finland; 2 Faculté de Médecine, Université Lyon-1, Lyon, France; 3 Forendo Pharma Ltd., Turku, Finland

**Keywords:** estrogen receptor, SERM, macrophages, inflammation, raloxifene, estradiol

## Abstract

Signaling *via* estrogen receptors (ER) is recognized as an essential part of the immune regulation, and ER-mediated signaling is involved in autoimmune reactions. Especially ERα activation in immune cells has been suggested to skew cytokine production toward Th2/M2-type mediators, which can have protective effect on inflammatory diseases and reduce Th1 and Th17 responses. These effects are caused by increased alternative activation of macrophages and changes in the activation of different T cell populations. In humans, hormonal status has been shown to have a major impact on several inflammatory diseases. Selective estrogen receptor modulators (SERMs) are ER ligands that regulate ER actions in a tissue-specific manner mostly lacking the adverse effects of steroid hormones. The impact of SERMs on the immune system is less studied, but it is suggested that certain SERMs may also produce immunoprotective effects. Here, we show that two novel SERMs and raloxifene affect immune cells by promoting M2 macrophage phenotype, alleviating NFκB activity, inhibiting T cell proliferation, and stimulating the production of anti-inflammatory compounds such as IL10 and IL1 receptor antagonist. Thus, these compounds have high potency as drug candidates against autoimmune diseases.

**Electronic supplementary material:**

The online version of this article (10.1007/s10753-018-0763-1) contains supplementary material, which is available to authorized users.

## INTRODUCTION

Signaling *via* estrogen receptors (ER) is recognized as an essential part of the immune regulation, and ER-mediated signaling involved in both chronic inflammatory diseases and autoimmune reactions [1–6]. This regulation can be either pro- or anti-inflammatory depending on several criteria such as types of organs and cells involved, source of immune stimulus, and variability of expression of ER subtypes in the cellular microenvironment [7]. Estrogenic compounds such as female sex hormones elicit their effects *via* ER. Upon ligand binding, ER initiates gene transcription in the nuclei and also elicits immediate effects *via* cytosolic signaling cascades. ER have been utilized as a drug target for several estrogen-regulated diseases, most importantly breast cancer and osteoporosis, in estrogen-sensitive organs [8]. However, ER-modulated inflammatory diseases and autoimmune reactions are not only limited to traditional estrogen target tissues.

Estrogenic signaling is suggested to affect immunomodulation in a wide array of inflammatory diseases such as intestinal inflammation and CNS autoimmunity [7]. ER ligands possibly induce anti-inflammatory effects *via* mechanisms involving ERα and GPER activation on immune cells, inducing a Th2-type skew in the cytokine milieu and reducing Th1 and Th17 responses [1, 9–13]. This anti-inflammatory shift includes increased M2 characteristics in monocyte macrophage populations and changes in the activity and number of T regulatory cells (Treg) [14–17]. It is intriguing that a similar Th2-type skew in inflammatory mediators has been observed to occur at the third trimester of pregnancy—a period also characterized by increased estrogen levels [18]. These observations suggest that ER signaling regulates the immune system cells by modulating their responses to inflammatory stimuli.

The activation of ER signaling is considered to stimulate anti-inflammatory response. Accordingly, 17β-estradiol (E2), a strong ER agonist steroid hormone, is associated with amelioration of inflammatory diseases [7]. E2 is not, however, utilized as an immunomodulatory drug because it may increase a risk for tumor formation in estrogen-sensitive tissues [19]. Therefore, it is not an optimal drug for long-term medication. Instead, a recent clinical trial suggests that another natural estrogen hormone, estriol, may be more suitable as an immune modulator. Estriol, the natural level of which is high especially during pregnancy, together with glatiramer acetate, has been shown to reduce the relapse rate of female MS patients [14, 20]. This result is supported by clinical findings showing that symptoms of multiple sclerosis are often alleviated during pregnancy [21].

Selective estrogen receptor modulators (SERMs) are compounds that elicit estrogenic and/or antiestrogenic effects in a tissue-specific manner. The first non-steroidal SERM developed for clinical use was tamoxifen, which has been used as an endocrine therapy for breast cancer since the late 1980s [22]. Thereafter, a few other SERM drugs such as raloxifene, toremifene, and ospemifene have been developed for treatment and/or prevention of various diseases in estrogen-responsive tissues. As the activation of ERs in immune cells has been linked with downregulation of inflammation, it is tempting to expect that an appropriate SERM might regulate the ER signaling in immune cells but not induce adverse effects in other estrogen-sensitive tissues.

Monocytes and macrophages are myeloid antigen-presenting cells that have a central role in the initiation of immune responses thus possessing a major role in adaptive immunity. In addition to phagocytosis, they can either promote or alleviate immune responses [23]. Currently, it is widely accepted that rather than just pro-inflammatory M1 and anti-inflammatory M2 dichotomy, the macrophages can adapt a spectrum of phenotypes with different patterns of cytokine production and receptor expression, according to their microenvironmental cues [24]. Several studies suggest that E2 activation modulates macrophage phenotype and function [12, 16, 25–28] although E2 does not elicit strong proliferative response in myeloid cells in a similar degree as in classical estrogen-sensitive tissues such as breast epithelium and endometrium [19]. Nevertheless, recent studies suggest that endogenous E2 may also induce macrophage activity and self-renewal *in vivo* [16, 26, 29]. The expression of ER in myeloid cells has been demonstrated, but the pattern of ER subtypes and splice variants in macrophages and their possible contribution to the innate immunity is likely to be context/tissue specific [16, 28, 30, 31].

We have studied the immune-modulatory properties of SERM drugs and several novel SERM candidates and examined their effects on NFκB activity and macrophage surface protein expression in human monocytes. Among those compounds, we have selected the most M2-type activation promoting compounds to be further studied in models of macrophage differentiation and cytokine production. Here, we present evidence that specific SERM compounds can modulate the function of the monocyte macrophage cell population. We show that specific SERMs can modulate the differentiation of CD14-positive cells by skewing their phenotype toward M2 macrophages and inducing the production of anti-inflammatory cytokines. These novel, previously unpublished SERM compounds promoted M2-like macrophage activation in pharmacological concentration. They could thus be beneficial for the alleviation of systemic inflammation in autoimmune diseases.

## MATERIALS AND METHODS

### SERMs

All SERM compounds, including novel compounds investigated in this study, were a kind gift from Forendo Pharma Ltd., Turku, Finland, unless stated otherwise. Raloxifene and 17β-estradiol were purchased from Sigma (St. Louis, USA). Working dilutions of estrogenic compounds were prepared in dimethylsulfoxide and kept at − 20 **°**C.

### Estrogen Receptor Affinity Assay

The binding affinity of SERM2, SERM7, and raloxifene to ERα and their ability to compete in binding with E2 (incubation time 2 h, estrogen concentration 2 nM) were determined using a commercially available assay kit according to the manufacturer’s instructions (PanVera LCC, Madison, WI) [32]. The IC50 values were obtained by fitting the data to the Hill’s equation [33].

### Cell Culture

Human THP-1 Lucia NFκB reporter cells were purchased from InvivoGen (San Diego, CA, USA). The THP-1 Lucia NFκB cell line contains a stable NFκB inducible Lucia reporter construct which is secreted to the growth medium. THP-1 Lucia cells were grown in a colorless RPMI-1640 growth medium with 4.5 g/l glucose, 10 mM HEPES, 1.0 mM sodium pyruvate, 2 mM L-glutamine, Pen-Strep (50 U/ml, 50 mg/l), 100 mg/l Normocin (InvivoGen), and 10% heat-inactivated fetal bovine serum (iFBS, EU approved). All reagents unless stated otherwise were purchased from Thermo Fisher Scientific Life Technologies (Carlsbad, CA, USA). Cells were maintained by passing them every 3–4 days by inoculating 0.5–1 × 10^6^ cells to fresh medium. To maintain selection pressure, every other passage was supplemented with 100 mg/l Zeocin (InvivoGen). All reagents used in cell culturing were purchased from Thermo Fisher Scientific Life Technologies (Carlsbad, CA, USA) unless stated otherwise.

MCF-7 and Ishikawa estrogen responsive element (ERE) Luc reporter cells were transfected with the reporter construct ERE Luc containing two tandem consensus ERE cloned into the pGL2-Promoter cloning vector. Cells were grown and maintained in DMEM medium supplemented with 10% iFBS, 2 mM L-glutamine, 150 μM G-418 for selective pressure, 10 μg/ml insulin, and 1 nM E2 (two latter ones only for MCF-7). Other methods were similar to Barsalou et al. [34] and Kallio et al. [35].

### Primary Cell Isolation and Differentiation

Peripheral venous blood was obtained from healthy males, and leukocytes were separated using a Ficoll-Paque (GE Healthcare Life Sciences, Uppsala, Sweden) solution. Peripheral blood mononuclear cells (PBMC) were collected and washed with PBS after centrifugation. Monocytes were isolated by CD14-positive selection using anti-CD14 magnetic beads (Miltenyi Biotech, Lund, Sweden). Monocytes were suspended in colorless αMEM supplemented with glutamine, penicillin–streptomycin, and 10% iFBS (EU). Monocytes were plated at 5 × 10^5^ cells per well in clear 12-well plates. Monocytes were cultured in fresh medium for 6 days at 37 °C to allow differentiation into macrophages. Resting cells were polarized into M1 or M2 type of macrophages by incubation for 6 days with interferon-γ (IFNγ, 50 ng/ml), interleukin-4 (IL4) (50 ng/ml), or IL10 (50 ng/ml) [36]. M(IFNγ) cells were activated with lipopolysaccharide (LPS, 10 ng/ml) after 5-day incubation with IFNγ.

Monocytes were treated with SERMs or E2 to assess their effects on macrophage polarization and phenotype. Compounds were added 4 days after the differentiation stimuli (IFNγ), followed by LPS addition 24 h later. After 6 days of culturing, medium samples were collected and cells were either lyzed for RNA extraction with RA1 lysis buffer (Macherey-Nagel, Duren, Germany) supplemented with β-mercaptoethanol or detached (with Accutase) for flow cytometry analysis. Cell mediums and lysates were kept at −80 **°**C prior to RNA or protein quantitation assays.

### Subjects

All subjects donating blood were volunteers, informed about the study, and they gave a written consent on the use of their cells. No personal information of the subjects was recorded. We used blood cells from total six healthy, non-obese males with age range 20–50 years. The experiments with human cells were done under approval of the University of Turku Ethics Committee (Statement Ref. 6/2017), and studies were conducted in accordance with the Helsinki Declaration.

### Cell Viability Assay

The cytotoxicity of SERMs on CD14+ and THP-1 Lucia cells was evaluated with the AlamarBlue cell viability reagent (Thermo Fischer) according to the manufacturers’ instructions. Briefly, cell viability was determined by culturing cells at least in triplicate in 100 μl of medium (5 × 10^5^ cells/well) for 2 days in flat-bottomed 96-well plates with respective treatment. After incubation, 10 μl of AlamarBlue reagent was added to each well and the microtiter plate further incubated for 2 h at 37 **°**C in 5% CO_2_. Then, the fluorescence was read with a HIDEX Plate Chameleon Reader (Hidex Ltd., Turku, Finland).

### Luciferase Reporter Activity Assays

THP-1 Lucia reporter cells were grown on 96-well plate (1 × 10^5^/well) 24 h in the presence of SERM followed by 24 h with LPS or tumor necrosis factor alpha (TNFα, Life Technologies) stimulation. Cell culture medium samples were collected for reporter activity assay, and cell viability was measured as described in the previous chapter. Cells were washed and freeze-thaw lyzed for total protein assay. Luciferase activity was determined by relocating 20 μl aliquots of cell culture media into optical Lumitrac 96-well plates (Greiner Bio-One, Krensmünster, Austria) followed by Quanti-Luc luciferase substrate (InvivoGen). Plates were read immediately for luciferase activity with Victor2 multiplate reader (PerkinElmer).

To study ERE Luc activation, MCF-7 and Ishikawa reporter cells were pre-incubated in colorless DMEM medium supplemented with charcoal-stripped serum to remove endogenous steroids without adding insulin or E2 [35]. Cells were plated at densities of 400,000 (Ishikawa) or 200,000 (MCF-7) per 96-plate well. After 24-h incubation the media were replaced with fresh ones with or without the study drugs and/or 1 nM of E2 dissolved in DMSO. After 48 h, media were again removed and replaced with DMEM. Fifty microliters of freshly reconstituted luciferase substrate was added into each well. After a 5-min incubation at room temperature, the plate was measured with Victor multilabel counter.

### RNA Extraction and Gene Expression Analysis

RNA was extracted from lyzed cell samples by using the Nucleospin RNA kit (Macherey-Nagel) according to the manufacturers’ instructions. Total RNA concentration in samples was determined by using Nanodrop ND-1000 (Nanodrop, Wilmington DE, USA). RNA sample was eluted in RNAse free water and translated to cDNA using Sensifast cDNA Synthesis Kit (Bioline, London, UK). Quantitative PCR was performed on a CFX96 thermal cycler (Bio-Rad Laboratories, Hercules, USA) with Taqman Gene Expression Assays (Applied Biosystems, Foster City, CA, USA). Eight nanograms of total RNA was used per sample unless stated otherwise, and the assay was performed according to the manufacturers’ instructions. The delta-delta Ct method was used for relative quantification of gene expression. Glyceraldehyde 3-phosphate dehydrogenase (GAPDH) was used as a reference gene. Taqman primer information is provided in supplementary Table 1.

### Flow Cytometry Analysis

Macrophages and monocytes were analyzed with the BD LSR Fortessa flow cytometer (BD Biosciences, Erembodegem-Dorp, Belgium). Cells were detached by Accutase (Life Technologies), washed with PBS with 0.1% FBS, and filtered through 35-μm pore cell-strainer snap caps (Corning Incorporation, Durham, NC, USA). Before staining, cells were transferred to a 96-well plate and Fc receptors were blocked with Human BD Fc Block reagent (BD Biosciences). Cells were then incubated 1 h with the fluorochrome-labeled anti-human monoclonal antibodies (from BioLegend, San Diego, CA, USA, unless stated otherwise: CD14 APC/Cy7 (clone63D3), CD163 Alexa 647 (clone GHI/61; BD Biosciences), CD192 BV605 (clone K036C2), and CD206 Alexa Fluor 488 (clone 15-2)). Cells were washed with PBS prior to analysis, and the expression of cell surface proteins was analyzed. Unstained macrophages were included as negative control for staining. Data were analyzed with Flowing Software (Turku Centre of Biotechnology, Turku, Finland), after gating on the myeloid population in the FSC/SSC plot with 10,000 events recorded. Results were expressed as the relative values of the median fluorescence intensity (MedFI) of each fluorochrome normalized to the respective value of unstained control. The percentage of cells expressing a specific phenotype was calculated according to the number of cells in the dot plot quadrants after gating.

### Macrophage Secretion Analysis

Concentrations of inflammatory regulators IL1β, IL10, chemokine (C-C motif) ligand 2 (CCL2), interleukin 1 receptor antagonist (IL1RA), and TNFα in cell culture medium were analyzed with multiplex assays (eBioscience Ltd., Cheshire, UK) according to manufacturer’s instructions by using the Luminex 200 system (Luminex Corporation, Austin, TX). Medium samples were collected after 6 days of culturing CD14-positive cells (500,000 cells in 1 ml of medium per sample) and kept at − 80 **°**C until analyzed. To remove debris, samples were centrifuged (5 min, 7000 rpm) prior to the assay. For IL1RA and CCL2 assays, samples were first diluted 1:100 in culture medium.

### T Cell Stimulation and Proliferation Assays

PBMCs were isolated from heparinized blood as described in the previous chapter. The cell suspension was diluted to a final concentration of 10^6^ cells/ml and plated in flat-bottomed 96-well culture plates, 1 × 10^5^ cells/well. Cells were activated with 10 μg/ml phytohemagglutinin (PHA-P) from *Phaseolus vulgaris* (Sigma-Aldrich, St. Louis, MO, USA). Plates were incubated for 3 days at +37 **°**C in 5% CO_2_. Non-adherent cell proliferation was measured by the thymidine incorporation method. After incubation, 1 μCi [6-^3^H]-thymidine (PerkinElmer, Boston, MA, USA) was added to each well, followed by 20 h of incubation. Cell suspensions were directly transferred to MultiScreen HTS FB 96-well filter plate (Merck Millipore, Darmstadt, Germany), and the wells were washed twice with Milli-Q-grade water and dried thoroughly. Optiphase HiSafe 3 (PerkinElmer, Waltham, MA) scintillation cocktail was added in the wells, and the plate was sealed. After 30-min incubation at RT, the plate was counted on a Wallac 1450 Microbeta Trilux β-counter (PerkinElmer, Turku, Finland) for 1 min per well.

## RESULTS

### Characterization of Two Novel SERMs

We characterized the abilities of two novel compounds (referred here as SERM2 and SERM7) to activate or inhibit ERE-mediated signaling in two estrogen-sensitive reporter cells and the compounds’ ability to compete for ER binding with E2 [37] [32]. As shown by the higher IC50 value, SERM2 bound to ERα with a slightly lower affinity than raloxifene and E2 (Table 1). The affinities of E2 and raloxifene to ERα were of similar magnitude to previously reported ones [38]. SERM7 binding affinity to both ERα was considerably weaker than that of raloxifene.

**Table 1:** Characterization of Estrogenic Properties of Novel SERMs in Comparison to Raloxifene and 17β-Estradiol

	Binding affinity to ERα	MCF-7 ERE Luc activity		Ishikawa ERE Luc activity	
		Agonism	Antagonism	Agonism	Antagonism
SERM2	+++	0	++	+	+++
SERM7	+	0	+	0	+
Raloxifene	++++	0	+++	0	+++
17β-Estradiol	++++	++++	***−***	++++	***−***

Incubation time and estrogen concentration were 2 h and 2 nM for affinity assays and 48 h and 1 nM for reporter assays, respectively

*++++* IC50 0.5–2 nM, *+++* IC50 < 10 nM, *++* IC50 < 100 nM, *+* IC50 < 1 μM, *0* no estrogenic effects

The human breast cancer cell line MCF7 and the human endometrial cancer cell line Ishikawa stably expressing ERE Luc reporter constructs (MCF-7 ERE Luc reporter and Ishikawa ERE Luc reporter cells, respectively) were used to study ERE-mediated transcriptional activation by the SERMs *in vitro*. Both of these cell lines express ERα and ERβ and are commonly used to study estrogenic signaling [39–41]. At the concentrations higher than 100 nM, SERM2 was found to activate ERE in Ishikawa cells acting as an agonist. In the presence of E2, SERM2 antagonized Ishikawa ERE Luc reporter activity, at the same order of potency as raloxifene (Table 1). An agonistic action of SERM2 was not found in MCF-7 ERE Luc reporter cell, but SERM2 antagonized ERE Luc activity in MCF7, although with a considerably higher IC50 value when compared to raloxifene. SERM7 displayed weak ER antagonism in both reporter cell lines, but no SERM7-induced agonism was detected in either of them.

### Selective Estrogen Receptor Modulators Suppress NFκB Activation

**Fig. 1:**
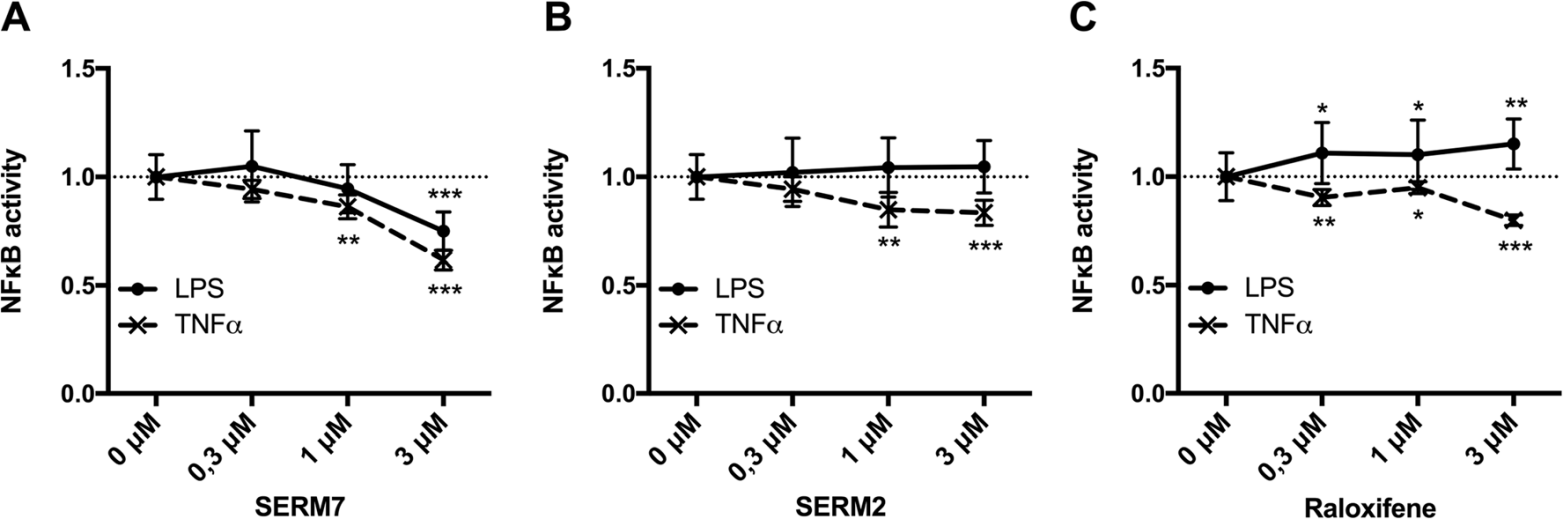
SERMs modulate NFκB activity in monocytic THP-1 Lucia reporter cells. Effects of **a** SERM7, **b** SERM2, and **c** raloxifene on 24-h LPS or TNF-induced reporter activity of THP-1 monocytes. c(LPS) = 10 ng/ml, c(TNFα) = 5 ng/ml. Dots and error bars represent mean ±SD. Statistical significance was determined after unpaired *t* test *vs* control (0 μM). *P < 0.05; **P < 0.01; ***P < 0.001.

**Fig. 2:**
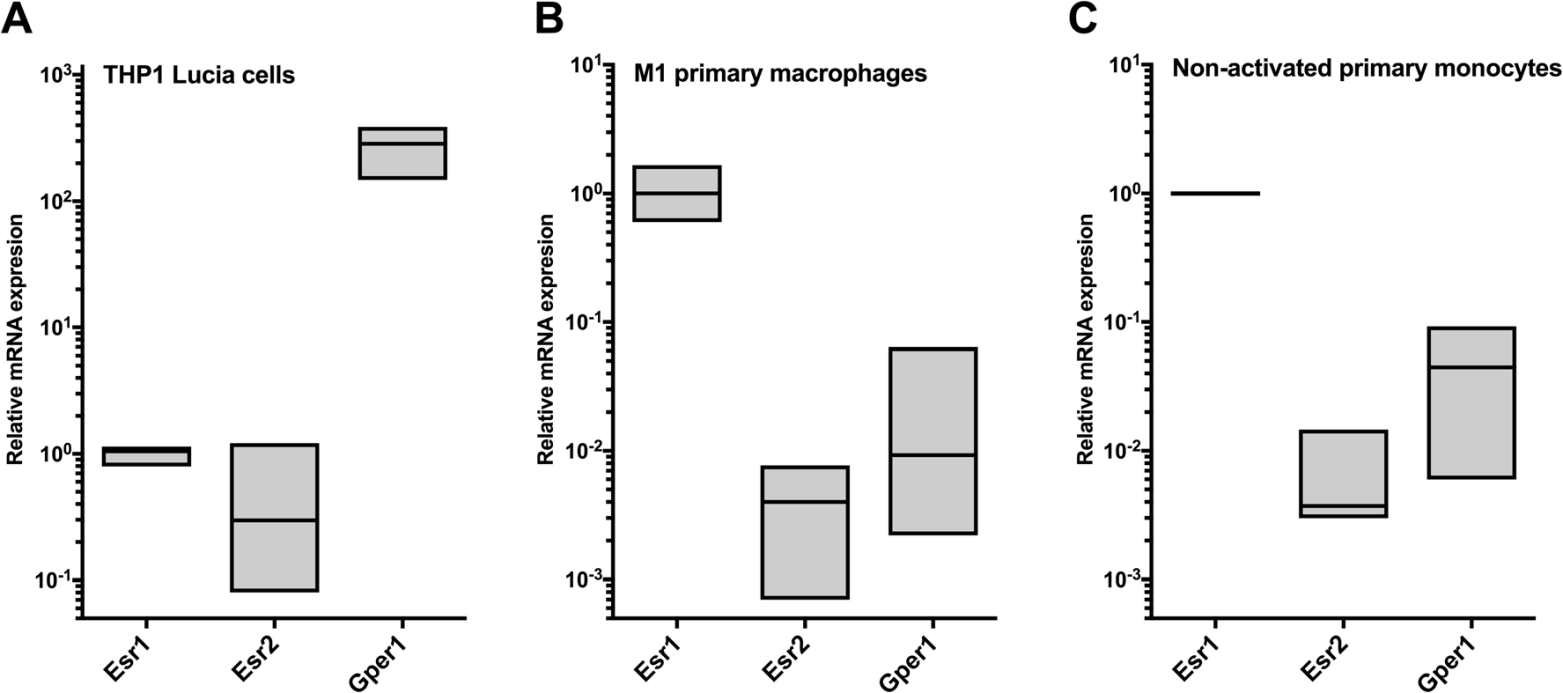
Expression of estrogen receptor subtypes (*Esr1* and *Esr2*) and *Gper1* genes in **a** THP-1 Lucia cells, **b** CD14-positive primary M(IFNγ+LPS) cells, and **c** non-activated monocytes. Boxes extend from min to max, and line in the middle represents median.

To elucidate the role of ERs on NFκB activation in monocytes, THP-1 Lucia cells with stable NFκB reporter expression were grown 24 h in the presence of SERM compounds, followed by 24 h with LPS or TNFα stimulation. First, several novel compounds and SERM drugs including tamoxifen, ospemifene, raloxifene, fulvestrant, and fispemifene were pre-screened for their NFκB modulatory activity, and the three most efficient ones were selected for a more specific analysis. The novel compound SERM7 inhibited both LPS- and TNFα-induced reporter activity in a concentration-dependent manner (Fig. 1a). Raloxifene and SERM2 also downregulated TNFα-stimulated, but not LPS-stimulated, reporter activity (Fig. 1b, c). In fact, raloxifene slightly increased LPS-stimulated activity. Both LPS and TNF alone produced an over 10-fold increase in reporter activity. Basal reporter activity was detectable, but none of the SERM compounds had significant effects on it (data not shown). *Esr1*, *Esr2*, and *Gper1* genes were all expressed in THP-1 Lucia cells (Fig. 2a).

### SERM Compounds Increase the M2 Phenotype Characteristics of CD14-Positive Macrophages

**Fig. 3:**
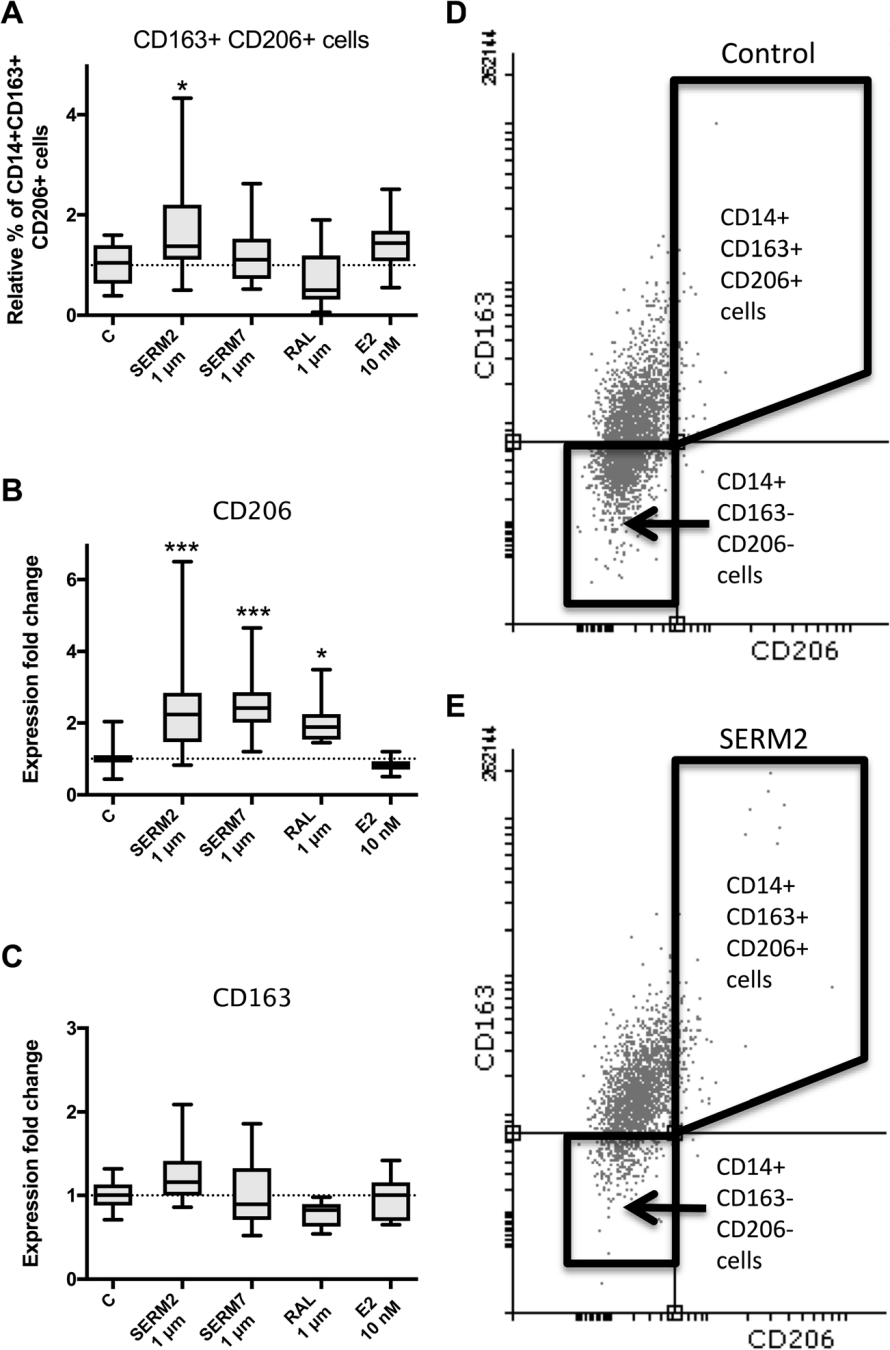
SERMs promote an alternative macrophage activation of monocytes by inducing activation of anti-inflammatory CD14+ CD163+ CD206 macrophage phenotype. **a** Proportion of CD14+ CD163+ CD206+ macrophages from CD14+ cell population. **b** Gene expression of CD206 and **c** CD163 in CD14+ M(IFNγ+LPS) cells. Box extends from the 25th to 75th percentiles; line in the middle represents median, and whisker from min to max. One-way statistical significance was determined after one-way ANOVA and Holm-Sidak post hoc multiple comparison test. *P < 0.05; **P < 0.01; ***P < 0.001. **d**, **e** Representative FACS dot plots (CD163-AF647 *vs* CD206-AF488) showing M1 (lower left quadrant) and double-positive CD163+ CD206+ (upper right quadrant) M2-like cells after IFNγ+LPS.

To further study whether the NFκB reporter attenuation was related to macrophage phenotypic changes in the M1-M2 axis, we measured the effect of SERMs on the macrophage surface marker expression by flow cytometry. To obtain macrophages of a specific phenotype, we cultured peripheral blood-derived CD14+ monocytes in the presence of IFNγ, IL4, or IL10. The expression of the *Esr1*, *Esr2*, and *Gper1* was confirmed by gene expression analysis, *Esr1* being over 10-fold higher to others in both M(IFNγ) cells and non-activated primary monocytes (Fig. 2b, c). Only a small percentage of IFNγ-induced macrophages express the surface proteins CD206 (mannose receptor, MRC-1) and CD163 compared to IL4- and IL10-stimulated monocytes (supplementary Figs. 1–2), and both CD163 and CD206 are considered strong M2 markers [17, 42]. Addition of 1 μM SERM2 to the culture, 24 h prior to LPS activation, increased the relative proportion of CD14+ CD163+ CD206+ macrophages (fold change 1.7; *P* = 0.025, Fig. 3a). This was supported by a significant elevation of CD206 mRNA expression (fold change 2.2; *P* < 0.001, Fig. 3b) and an observed increase in CD163 mRNA expression although not statistically significant (fold change 1.2; *P* = 0.12, Fig. 3c). Other studied SERMs did not have an effect on relative CD14+ CD163+ CD206+ cell number, but 17β-estradiol (E2) at 10 nM concentration caused (statistically not significant) stimulation but to a lesser extent than SERM2.

**Fig. 4:**
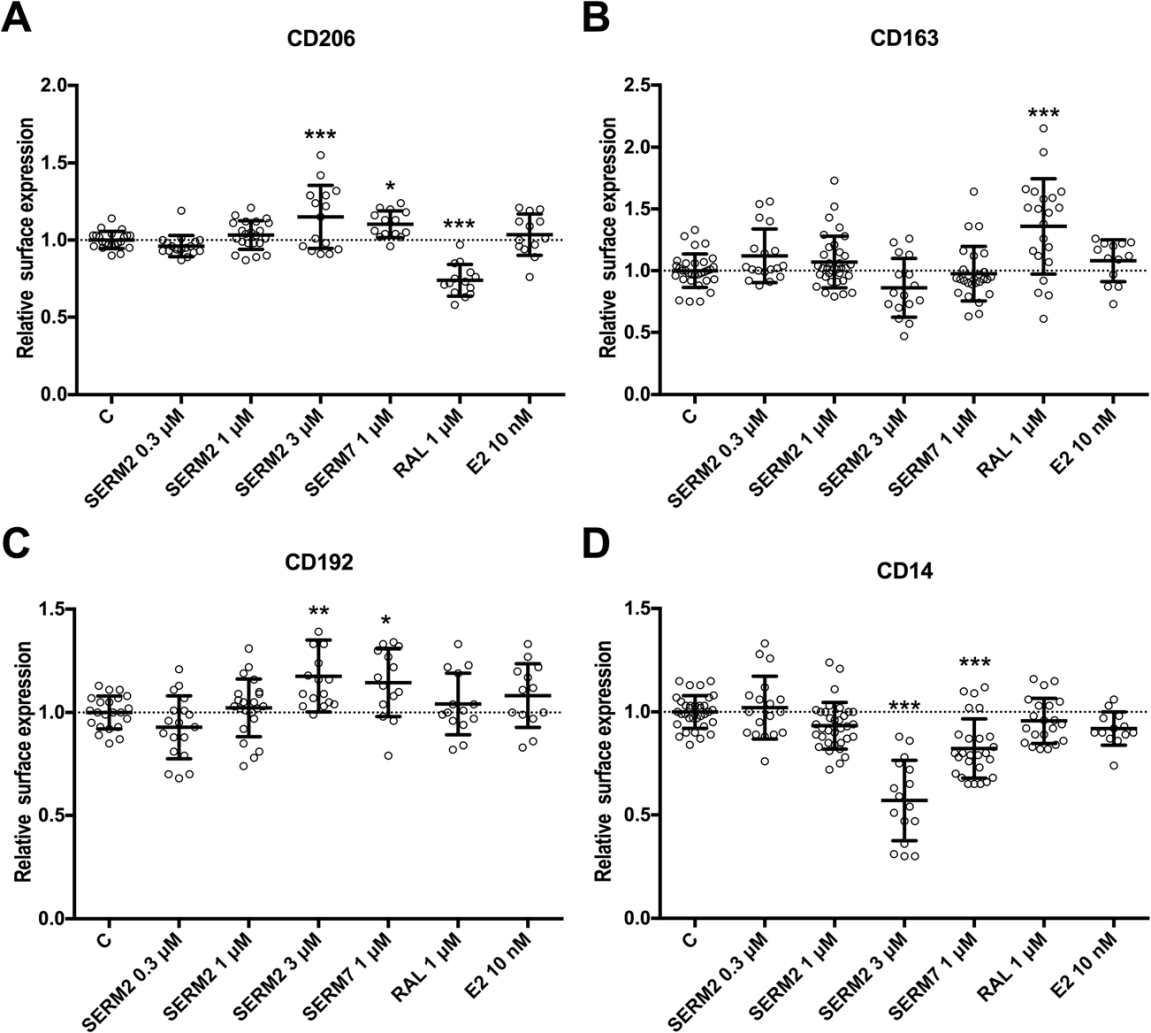
Effect of 48-h SERM/E2 treatment on median fluorescence intensity representing surface receptor expression of **a** CD206, **b** CD163, **c** CD192, and **d** CD14 in human-derived CD14-positive mononuclear cells, cultured 6 days for monocyte polarization and IFNγ 50 ng/ml followed by LPS activation at day 5. Scatter plot of individual samples with means ± SD. Statistical significance *vs* control group was determined after one-way ANOVA followed by post hoc Holm-Sidak multiple comparison test. *P < 0.05; **P < 0.01; ***P < 0.001.

Raloxifene and SERM7 both increased the gene expression of CD206 but not of CD163 (Fig. 3b, c). Nevertheless, raloxifene increased surface protein expression of scavenger receptor CD163 as demonstrated by its increased median fluorescence intensity (MFI) but reduced CD206 MFI (fold changes 1.4; *P* < 0.001 and 0.7; *P* < 0.001, respectively, Fig. 4a, b). Under the same point of observation, the level of CD206 mRNA was, however, increased (fold change 2.0; *P* = 0.025, Fig. 3b). We also studied the expression of a few other macrophage subtype-associated surface receptors. CD192 (CCR2) is considered to be an M2 marker, and both SERM2 and SERM7 increased moderately its MFI (Fig. 4c) whereas they significantly inhibited expression of CD14 surface protein (fold change 0.6 and 0.8, respectively, *P* < 0.001 for both, Fig. 4d) that is a component of the membrane protein complex responsible for pathogen detection and LPS-triggered pro-inflammatory responses [43]. E2 treatment also had a reducing trend on mean CD14 expression (Fig. 4d) (Table 2).

**Table 2:** Summary of the Statistically Significant Effects of SERM2, SERM7, and Raloxifene on Macrophage Polarization and Activity Markers, Presented in Figs. 1, 2, 3, 4, 5, 6, and 7

	SERM2	SERM7	Raloxifene
NFκB activity (LPS-induced)	n	***−***	+
NFκB activity (TNFα-induced)	***−***	***−***	***−***
Number of CD163+ CD206+ double-positive cells	+	n	n
CD14 surface expression	***−***	***−***	n
CD163 surface expression	n	n	+
*CD163* gene expression	n	n	n
CD192 surface expression	+	+	n
CD206 surface expression	+	+	***−***
*MRC1* gene expression	+	+	+
*IL10* expression	+	n	+
IL10 secretion	+	n	n
*IL1RN* expression	+	+	n
IL1RA secretion	n	n	n
*CCL2* expression	n	***−***	***−***
CCL2 secretion	n	n	n
PHA-activated T cell proliferation	***−***	***−***	***−***

*+* significant increase, − significant decrease, *n* no effect

### Novel Selective Estrogen Receptor Modulators Induce the Production of Th2-Type Cytokines IL10 and IL1RA

**Fig. 5:**
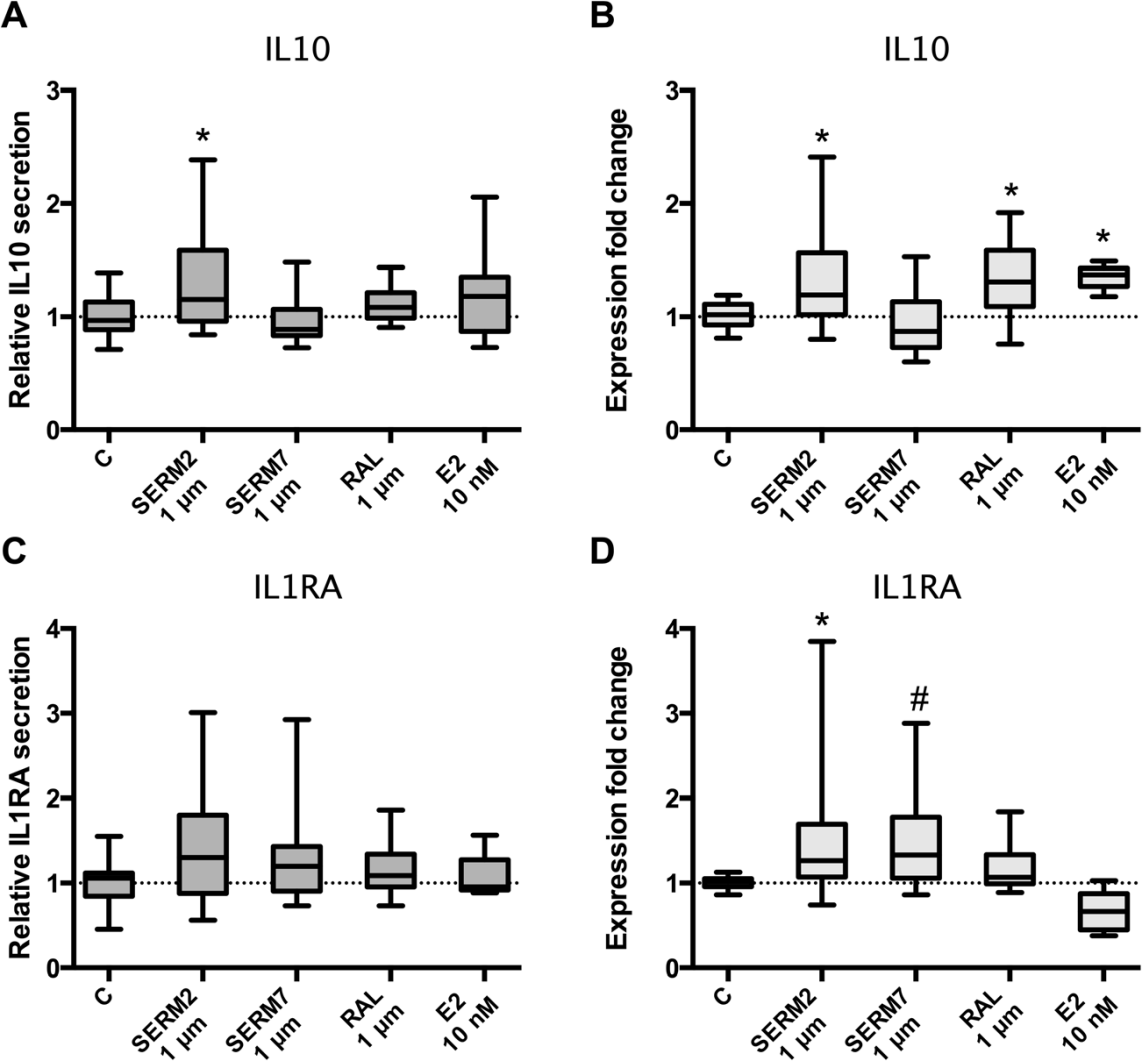
Estrogen receptor ligands modulate cytokine synthesis and secretion of (IFNγ+LPS)-activated CD14+ cells toward anti-inflammatory phenotype. **a** Secretion and **b** gene expression rates of IL10. **c**, **d** Respective values for IL1RA. Five hundred thousand cells per sample. Box extends from the 25th to 75th percentiles and whiskers from min to max. Statistical significance was determined after one-way ANOVA followed by post hoc Holm-Sidak multiple comparison test. ^#^*P* ≤ 0.1; *P < 0.05.

After the flow cytometric studies, we wanted to assess whether the cytokine production of CD14+ M(IFNγ) cells can also be skewed toward the Th2/M2 phenotype by SERM treatment. We found that 1 μM SERM2 and raloxifene and 10 nM E2 increased IL10 mRNA expression in M(IFNγ) cells (fold change 1.3 for all; *P* = 0.026, 0.041, and 0.05, respectively, Fig. 5a). Concomitantly, IL10 protein secretion from cells was increased as demonstrated by multiplex analysis from cell growth media, SERM2 having the most significant effect on IL10 secretion (fold change 1.4; *P* = 0.014, Fig. 5b). In addition to IL10, the gene expression of IL1RA was upregulated after SERM2 (fold change 1.5; *P* = 0.05, Fig. 5c) and SERM7 (fold change 1.4; *P* = 0.05) treatments. Mean IL1RA protein secretion rate was increased, respectively, but these results failed to reach the significance due to considerable variation (fold change 1.4; *P* = 0.19 for SERM2, Fig. 5d).

### SERM-Induced Macrophage Polarization Toward a M2 Phenotype Does Not Inhibit Inflammatory Signaling

**Fig. 6:**
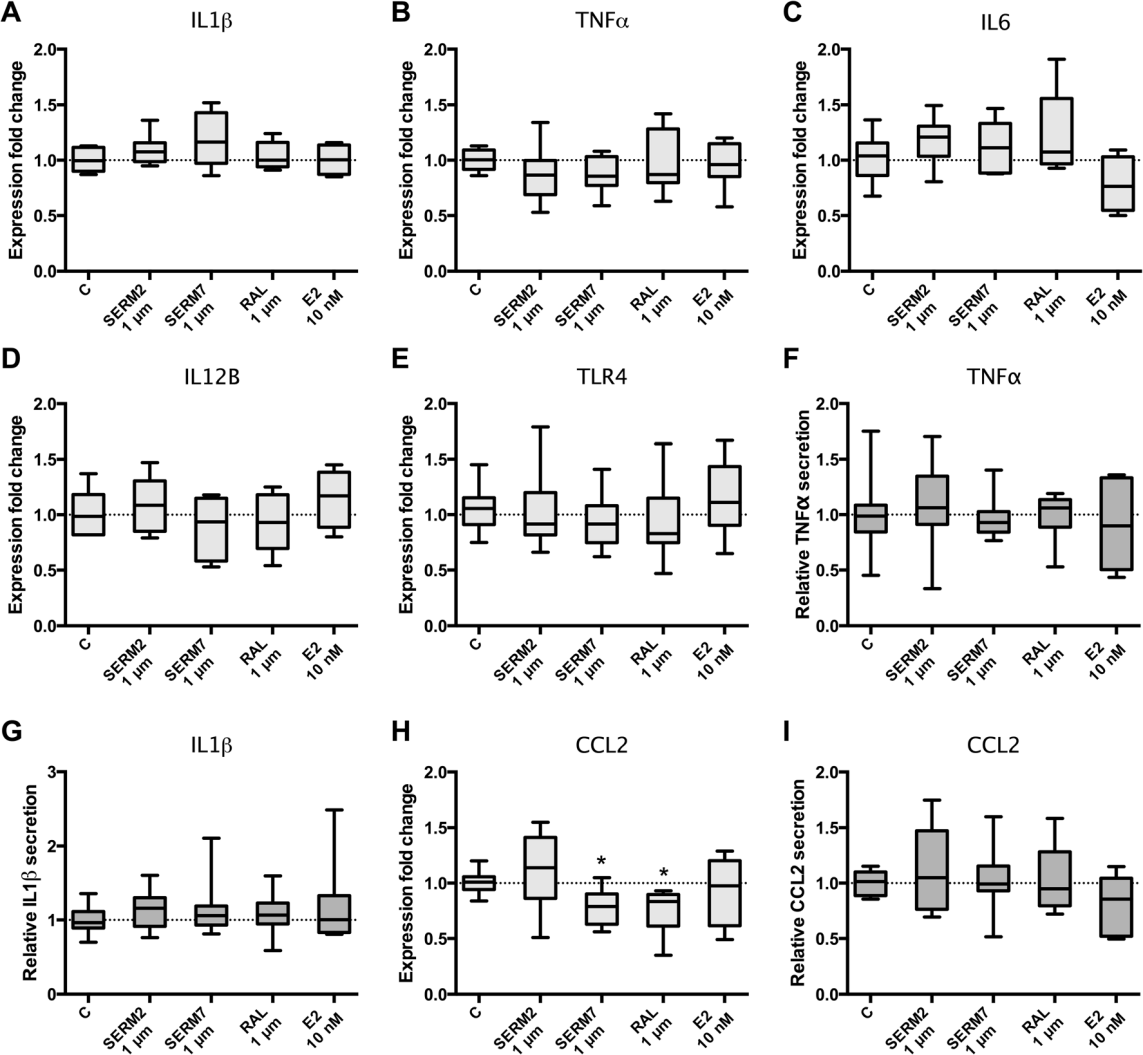
Estrogen receptor ligands do not downregulate proinflammatory signaling in (IFNγ+LPS)-activated CD14+ macrophages. Gene expression rates of **a** IL1β, **b** TNFα, **c** IL6, **d** IL12B, and **e** TLR4. Secretion rates of **f** TNFα and **g** IL1β from M(IFNγ+LPS) cells. **h** Gene expression and **i** secretion rates of CCL2. Five hundred thousand cells per sample. Box and whisker as min and max. Statistical significance was determined after one-way ANOVA followed by post hoc Holm-Sidak multiple comparison test. *P < 0.05.

As our results suggested that SERMs skew macrophage phenotype toward M2 (Figs. 3, 4, and 5), we were interested in whether expression of the genes for and secretion of pro-inflammatory mediators is attenuated to same extent. Neither SERMs nor E2 affected the expression of the pro-inflammatory genes IL1β, TNFα, IL6, IL12B, and TLR4 in LPS-activated M(IFNγ) cells (Fig. 6a–e). Respectively, secretion of IL1β and TNFα from CD14+ cells to growth medium was not changed (Fig. 6f, g). The only gene responsive to ER ligands and considered to be pro-inflammatory was CCL2, whose expression was moderately downregulated by SERM7 and raloxifene (fold change 0.8 for both). Nevertheless, the attenuation of gene expression was not translated to protein secretion from the same cells (Fig. 6h, i).

### SERMs Inhibit T Cell Proliferation Rate

**Fig. 7:**
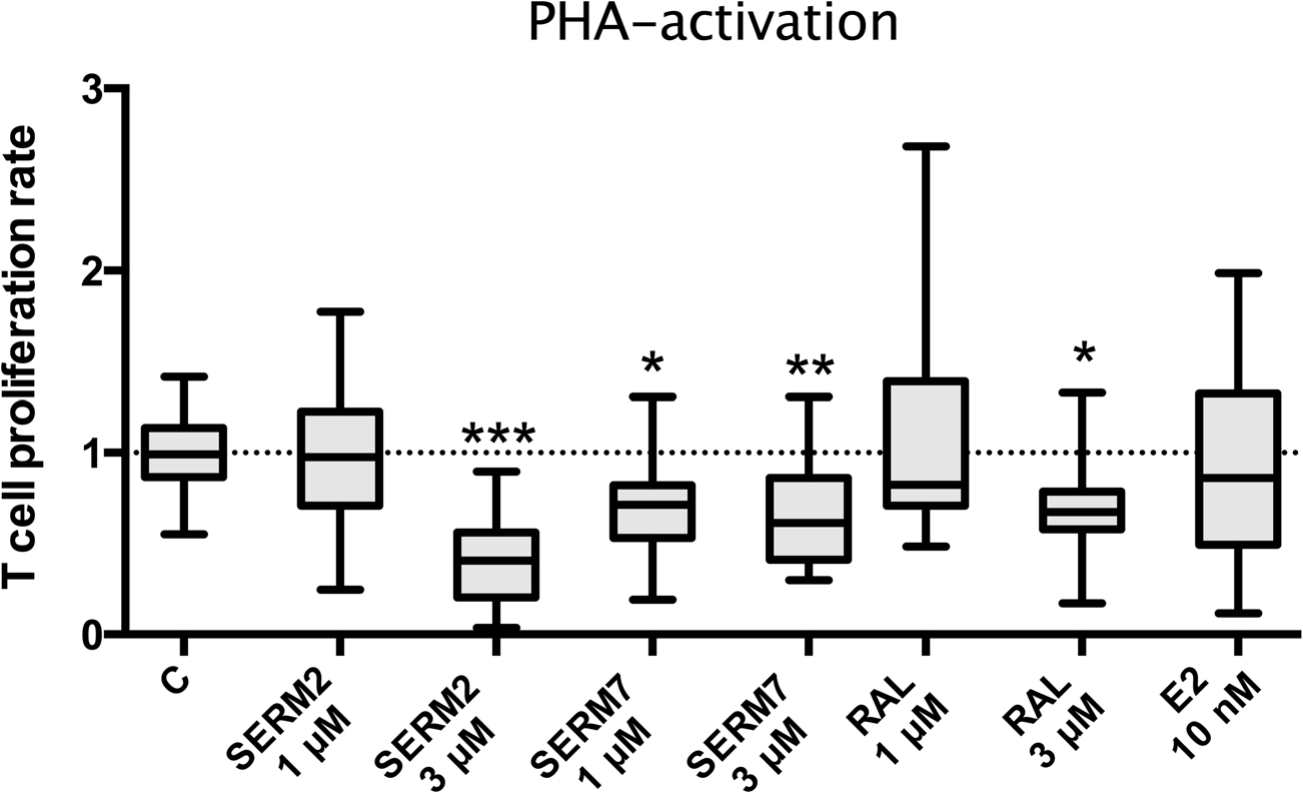
SERMs inhibit proliferation of PHA-activated primary T cells. Peripheral blood-derived mononuclear cells from five donors were cultured 3 days with phytohemagglutinin (PHA) and SERM or E2. Proliferation rate of non-adherent cells was measured with β-counter after 20 h ^3^H-thymidine incorporation. Selective estrogen receptor modulator stimulates IL-10 secretion from IFNγ+LPS-activated CD14-positive monocytes. Box extends from the 25th to 75th percentiles; line in the middle represents median, and whisker from min to max. Statistical significance was determined after one-way ANOVA followed by post hoc Holm-Sidak multiple comparison test. *P < 0.05; **P < 0.01; ***P < 0.001.

To assess whether the SERM-induced macrophage polarization affected T cell activation, we measured the rate of T cell proliferation in co-cultures with PBMC. T cell proliferation was activated with the exogenous superantigen PHA, followed by a 48-h SERM treatment. SERM2, SERM7, and raloxifene all inhibited proliferation of activated T cells (fold changes 0.4, 0.7, and 0.7, respectively, Fig. 7). SERM7 suppressed proliferation rate at lower those than other SERMs here. The mean proliferation rate of E2-treated cells was below control, but there were substantial differences in E2 response between the cells from different donors. Interestingly, corresponding donor-associated effects were not found with other treatments or assays. SERMs (0.3–3 μM) and E2 (10 nM) alone at the concentrations used did not possess cytotoxic or growth stimulatory effects on T cells nor monocytes as cell viabilities were not changed by treatments (data not shown).

## DISCUSSION

Here, we present two new SERM-type compounds, which promote alternative M2 activation of myeloid cells. According to our results, the macrophage phenotype-polarizing anti-inflammatory activity of these new compounds is higher than those of previous SERM drugs. Macrophages and monocytes play a critical role in the pathogenesis of several autoimmune diseases by acting as key producers of mediators of inflammation further supporting the development of CD4+ and CD8+ pro-inflammatory cells [44]. The dysregulation of macrophages is found in various tissue-specific pathologies such as neuronal degeneration in Alzheimer’s disease and hyperplasia in synovial joints in rheumatoid arthritis [45, 46].

This study provided several lines of evidence that SERM7, raloxifene, and most efficiently SERM2 promoted M2-type characteristics in IFN + LPS-activated macrophages. First, the capacity of SERM2 to increase the number of CD14+ CD163+ CD206+ cells clearly suggests that a proportion of IFNγ-polarized macrophages were able to adopt a more anti-inflammatory phenotype even in the presence of LPS. There was a concomitant increase in the gene expression of the M2 markers after SERM2 treatment. We found that different SERMs activate M2 markers in to a variable degree, as raloxifene was the most potent CD163 activator whereas SERM2 and SERM7 significantly upregulated CD206 and CD192. Second, SERM2 and raloxifene increased the expression and secretion of the anti-inflammatory mediators IL10 and IL1RA that are suggested to inhibit pro-inflammatory signaling, antigen presentation, and the CD4+ effector T cell activation. IL10 and IL1RA are considered to be anti-inflammatory mediators whose synthesis and secretion are associated with the M2-subtype macrophages (Supplementary Fig. 3) [42, 47]. Third, SERM-induced macrophages suppressed T cell proliferation after antigen activation in primary PBMC culture. Fourth, SERM2, SERM7, and raloxifene suppressed the TNFα-induced NFκB activation of the reporter monocytes. Our data demonstrates that the selected SERMs contribute to M1/M2 macrophage activation by promoting anti-inflammatory properties in M1-like cells thus stimulating production of Th2-type compounds.

Our results are in line with the current perspective that, instead of the simplified M1/M2 dichotomy, macrophage polarization includes numerous states of activation with diverse functions [24, 48]. Interestingly, we did not find distinct evidence that the SERMs tested would directly downregulate pro-inflammatory cytokine signaling in CD14+ cells. In the cells where IL10 and IL1RA were upregulated, the expression and secretion of IL1β and TNFα remained unaltered. To confirm this result, we also measured the expression of a few additional inflammatory genes such as IL12B, IL6, and TLR4, but no SERM-induced downregulation was found either. CCL-2 was an exception among pro-inflammatory genes as its expression was downregulated by SERM7 and raloxifene. SERM2 has the most significant stimulatory effect on M2 markers, but its effect on M1 markers was very modest, and on the contrary to other SERM7 and raloxifene, it did not affect LPS-induced NFκB activity. LPS induction here is model for bacterial endotoxin-activated inflammatory response [49], and therefore, it is tempting to speculate that SERM2 might suppress autoimmune reactions but still not disrupting the innate immunity against pathogens.

Intriguingly, raloxifene stimulated LPS-induced but inhibited TNFα-mediated NFκB activation. Raloxifene treatment had also different trend between protein and mRNA expression of M2 surface markers in LPS-activated macrophages. Nonetheless, it has been previously presented that, on top estrogenic effects, raloxifene also mediates ER-independent reactions in CD14+ cells and may interfere their differentiation [50, 51] thus possibly enabling apparently ambivalent changes in cell phenotype. We will warrant the future *in vivo* studies to investigate which SERM is the most efficient in polarizing macrophages and inducing beneficial outcome in autoimmune diseases.

It is noteworthy that SERM2 induced M2 phenotype more effectively than E2, considering that estrogenic hormones in general are described in various studies mostly as anti-inflammatory compounds, reviewed, e.g., by Straub [7] and Villa et al. [52]. To date, there has been several questions concerning the mechanisms of the anti-inflammatory action of estrogen hormones that still remain unanswered [7, 52]. In addition to the general “anti-inflammatory tone” in which pro-inflammatory signaling is downregulated, E2-induced ER activation has been recently associated with polarization of murine macrophages [16, 25, 26]. However, the results reported in the literature suggest a diverse, context-specific role for E2 in macrophage activation. E2 promoted the M1 phenotype in rat alveolar NR8383 macrophages and aggravated joint inflammation [25], but on the other hand, ERα was required for alternative macrophage activation, and E2 treatment of primary monocytes made them more prone to adopt M2 phenotype [16]. E2 also accelerated inflammatory response in RAW264.7 cells by reducing the M1 > M2 transition time after LPS shock [26] and interfered LPS activation by impairing NFκB activation [17]. Our results are in line with these partly conflicting data of the E2 having diverse, context, or even donor-specific effects on immune system responses. We found that the effect of E2 on CD14+ cells varied from modest to non-existent and E2 induction on the proliferation rate of activated T cells varied significantly between the cells from different donors. Similar variation was not found among SERM-stimulated cell samples. A recent study suggests that endogenous E2 triggers macrophage proliferation in ovariectomized mice [29]. Nevertheless, we did not find E2 to modulate M1 type macrophage proliferation *in vitro*.

Besides E2, SERM drugs such as tamoxifen, fulvestrant, toremifene, and raloxifene have been previously linked with the inhibition of tissue macrophage-mediated inflammation and monocyte differentiation and protection against neural injuries [50, 53–57]. Our results support the idea that although some SERMs, such as raloxifene and tamoxifen, may also promote an anti-inflammatory response, the novel compounds SERM2 and SERM7 are more active in myeloid cells. However, the actual agonist/antagonist role of these SERMs on ERs and GPER in immune cells cannot be determined on the basis of our results. Previous studies lack unanimous results about contribution of various ERs on macrophage polarization, suggesting that activation of ERα or ERβ or even the membrane-associated GPER might alleviate macrophage-related inflammation [11, 58, 59]. We propose that ERα signaling may have the most crucial role in M2 polarization. We base our claim on the facts that SERM2, SERM7, and raloxifene all had significant binding affinity to ERα studied concentrations and ERα gene expression was the highest of ER subtypes in both resting and IFNγ/LPS-activated monocytes, over 100-fold higher to ERβ. THP-1 Lucia cells expressed somewhat different ER profile to primary CD14+ cells, *Gper1* being the most expressed ER. This difference is likely due to leukemic origin of THP-1 line, and we cannot exclude that NFκB reporter activity assays here might be also affected by abundant GPER signaling. Nonetheless, the central role of ERα in myeloid cell polarization and differentiation is suggested by other studies although the lack of immune cell-specific methods somewhat hampers the results [16, 27, 60].

There is an urgent need for new therapies for autoimmune diseases as their global prevalence is approximately 5% in the total population thus affecting far over 100 million people worldwide [61]. Novel potent drug molecules, especially cost-effective and well-tolerated, are highly required. Current SERM drugs lack several of the adverse effects of steroid hormones, and SERMs have been already used for decades as preventive and therapeutic agents thus providing plausible experience for prolonged usage. We present here three SERMs exerting anti-inflammatory effects in the myeloid cell microenvironment thus having a potential as novel pharmaceuticals to alleviate effects of chronic inflammation or autoimmune diseases. Further understanding the impact of SERMs on myeloid cell biology may also provide new insights into the benefits and disadvantages of ER-modulating drugs currently used for, e.g., estrogen-sensitive cancers. Our results are still preliminary, but we intend to validate these observations in appropriate animal studies. Our aim is to discover the most potent SERM to alleviate macrophage dysregulation in chronic inflammation and autoimmune diseases.

## Electronic Supplementary Material

Supplementary Figure 1 The relative proportion of triple positive CD14+ CD163+ CD206+ cells after activation with IFNγ+LPS, IL4 or IL10. Untreated monocyte (M0) were cultured 6-days similarly to activated. Box extends from min to max, line in the middle represents median. (GIF 17 kb).

High Resolution Image (TIFF 1081 kb).

Supplementary Figure 2 Effect of IFNγ+LPS, IL4 or IL10 activation on median fluorescence intensity of A) CD206, B) CD163, C) CD192, D) CD14 representing surface receptor expression in human derived CD14-positive mononuclear cells, cultured six days during the polarization. Untreated monocytes are marked as M0. Scatter plot with means±SD (GIF 60 kb).

High Resolution Image (TIFF 3597 kb).

Supplementary Figure 3 Effect of IFNγ+LPS, IL4 or IL10 activation on gene expression of A) CD206, B) CD163, C) IL10, D) IL1RA, E) IL1β, F) TNFα, G) CCL2, H) IL6, I) IL12B in human derived CD14+ mononuclear cells after six days of culturing. Untreated monocytes are marked as M0. Box represents min to max, line represents median (GIF 88 kb).

High Resolution Image (TIFF 4290 kb).

ESM 1 (DOCX 16 kb).
