# Supplementary figures and images for: SERMs Promote Anti-Inflammatory Signaling and Phenotype of CD14+ Cells
Source: Inflammation. 2018 Mar 24;41(4):1157–71. doi: 10.1007/s10753-018-0763-1 (PMC6061028; doi:10.1007/s10753-018-0763-1)

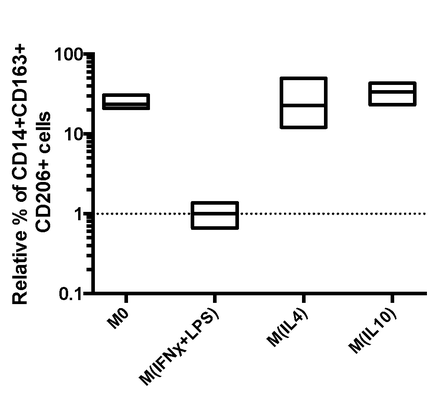

Supplement: Supplementary file 1 — The relative proportion of triple positive CD14+ CD163+ CD206+ cells after activation with IFNγ+LPS, IL4 or IL10. Untreated monocyte (M0) were cultured 6-days similarly to activated. Box extends from min to max, line in the middle represents median. (GIF 17 kb). [file 10753_2018_763_Fig8_ESM.gif]

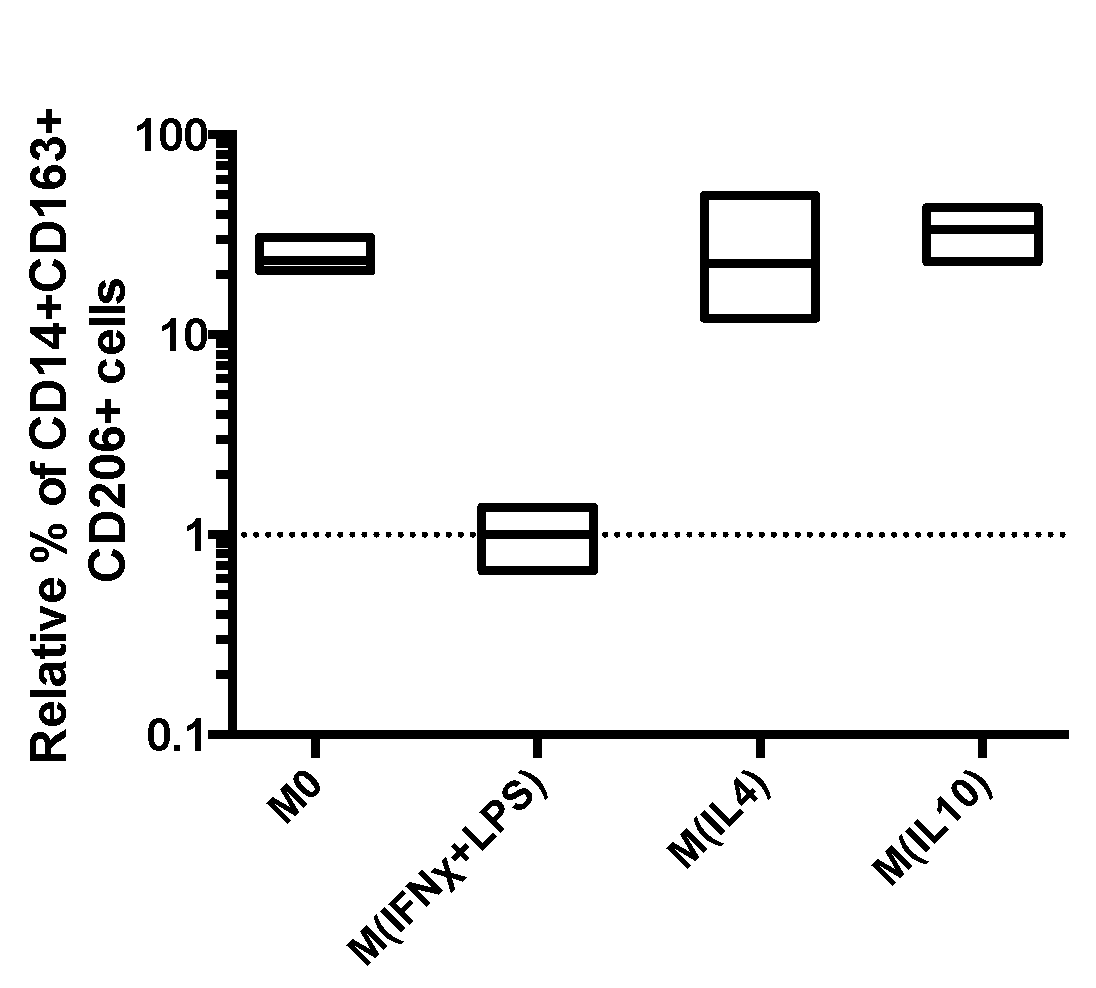

Supplement: Supplementary file 2 — High Resolution Image (TIFF 1081 kb). [file 10753_2018_763_MOESM1_ESM.tiff]

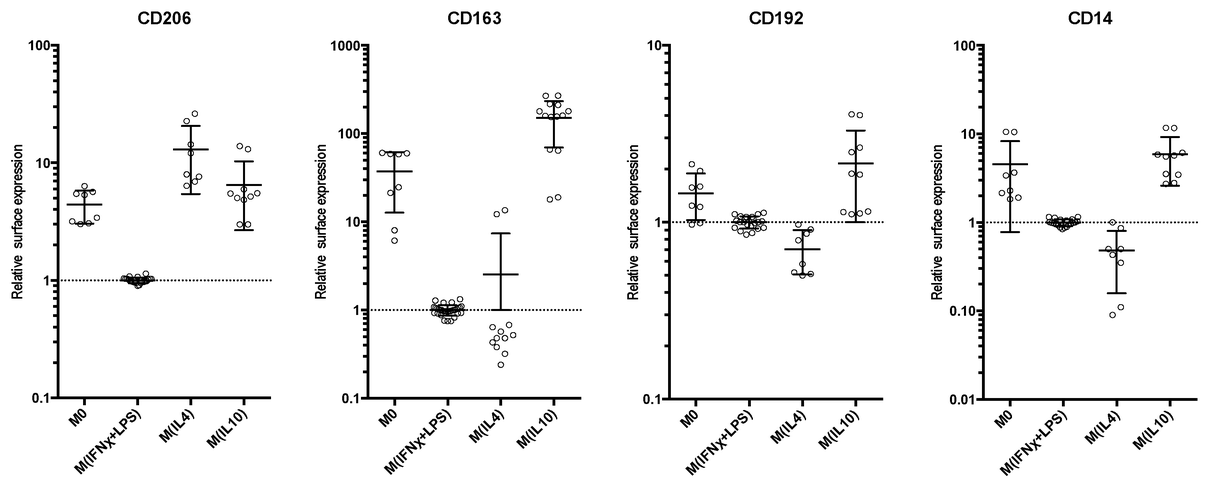

Supplement: Supplementary file 3 — Effect of IFNγ+LPS, IL4 or IL10 activation on median fluorescence intensity of A) CD206, B) CD163, C) CD192, D) CD14 representing surface receptor expression in human derived CD14-positive mononuclear cells, cultured six days during the polarization. Untreated monocytes are marked as M0. Scatter plot with means±SD (GIF 60 kb). [file 10753_2018_763_Fig9_ESM.gif]

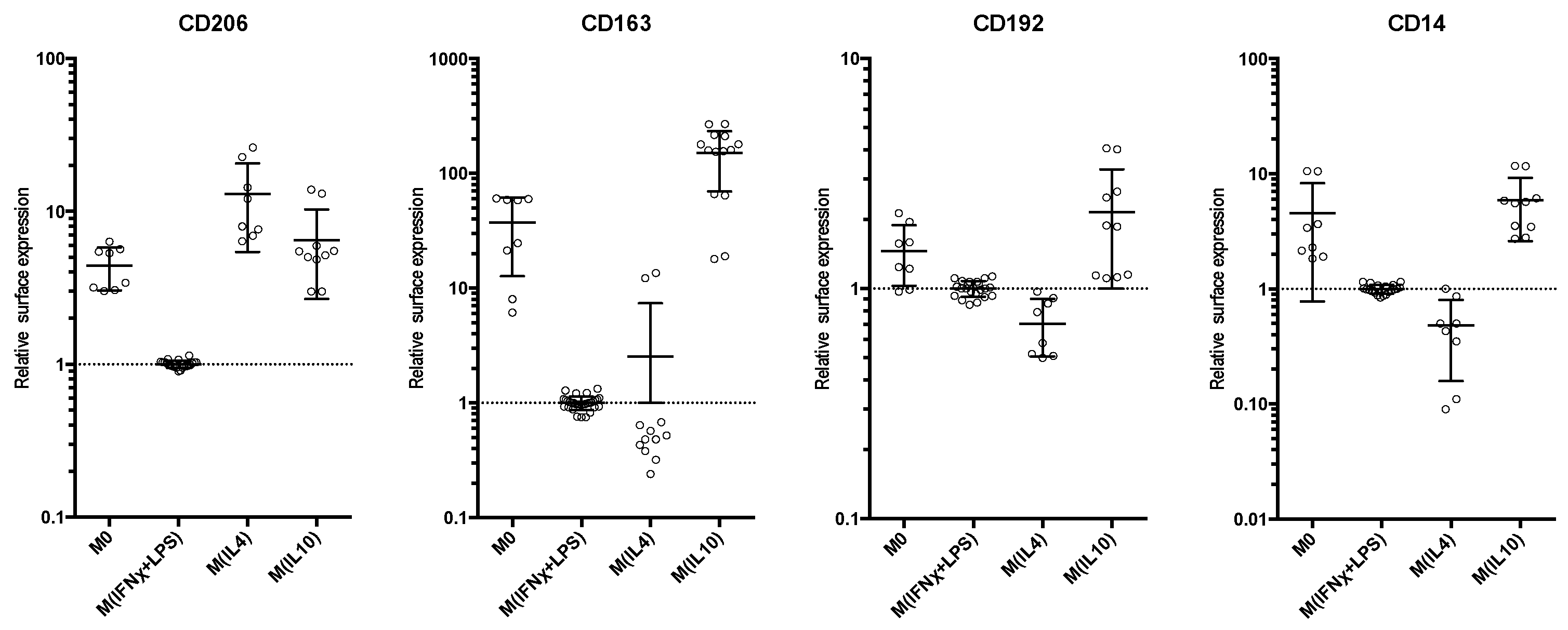

Supplement: Supplementary file 4 — High Resolution Image (TIFF 3597 kb). [file 10753_2018_763_MOESM2_ESM.tiff]

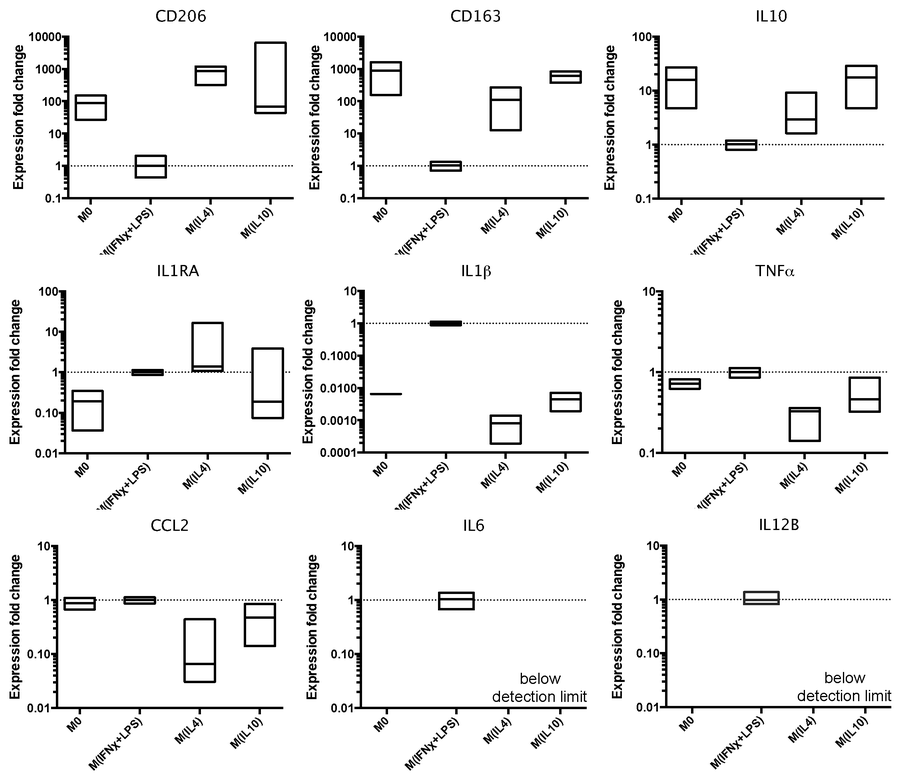

Supplement: Supplementary file 5 — Effect of IFNγ+LPS, IL4 or IL10 activation on gene expression of A) CD206, B) CD163, C) IL10, D) IL1RA, E) IL1β, F) TNFα, G) CCL2, H) IL6, I) IL12B in human derived CD14+ mononuclear cells after six days of culturing. Untreated monocytes are marked as M0. Box represents min to max, line represents median (GIF 88 kb). [file 10753_2018_763_Fig10_ESM.gif]

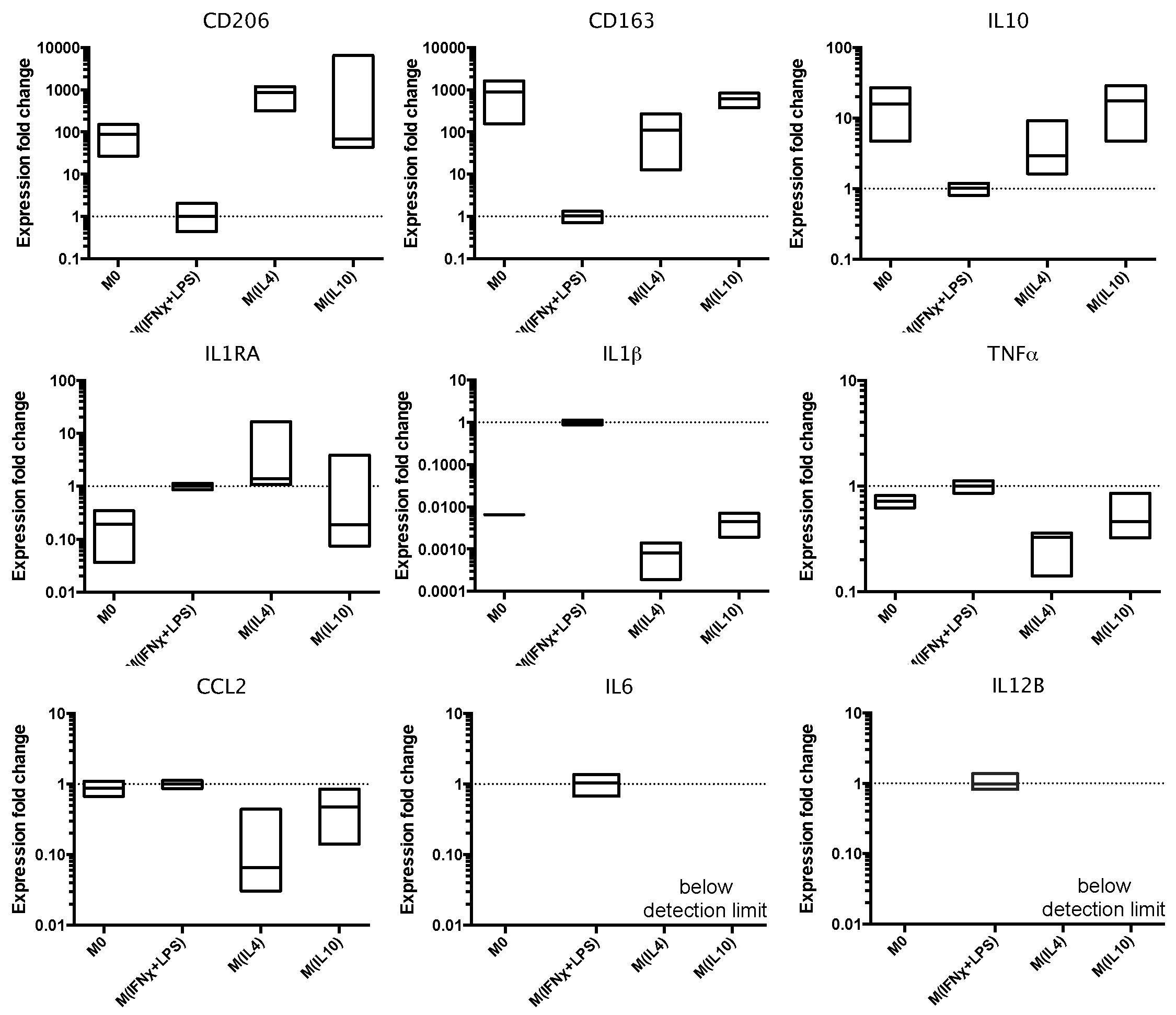

Supplement: Supplementary file 6 — High Resolution Image (TIFF 4290 kb). [file 10753_2018_763_MOESM3_ESM.tiff]
