# Supplementary material for: SERMs Promote Anti-Inflammatory Signaling and Phenotype of CD14+ Cells
Source: Inflammation. 2018 Mar 24;41(4):1157–71. doi: 10.1007/s10753-018-0763-1 (PMC6061028; doi:10.1007/s10753-018-0763-1)
Supplement: Supplementary file 7 — (DOCX 16 kb). [file 10753_2018_763_MOESM4_ESM.docx]

**Supplementary table caption** List of Taqman gene expression assays (Thermo Fisher Scientific) used in qPCR analysis

| *Analyte* | *Gene* | *Assay ID* |
| --- | --- | --- |
| Interleukin 1 beta | *IL1B* | Hs00174097_m1 |
| Interleukin 6 | *IL6* | Hs00985639_m1 |
| Interleukin 10 | *IL10* | Hs00961622_m1 |
| Interleukin 12B | *IL12B* | Hs01011518_m1 |
| Tumor necrosis factor alpha | *TNF* | Hs01113624_g1 |
| Estrogen receptor alpha | *ESR1* | Hs00174860_m1 |
| Estrogen receptor beta | *ESR2* | Hs00230957_m1 |
| G protein-coupled estrogen receptor 1 | *GPER* | Hs01922715_s1 |
| Actin beta | *ACTB* | Hs99999903_m1 |
| C-C motif chemokine ligand 2 | *CCL2* | Hs00234140_m1 |
| CD163 molecule | *CD163* | Hs00174705_m1 |
| Interleukin 1 receptor antagonist | *IL1RN* | Hs00893626_m1 |
| Toll like receptor 4 | *TLR4* | Hs00152939_m1 |
| Mannose receptor C-type 1 | *MRC1* | Hs00267207_m1 |
| Arginase 1 | *ARG1* | Hs00968979_m1 |
